# Cardioprotective Effect of Olive Oil Against Ischemia Reperfusion-induced Cardiac Arrhythmia in Isolated Diabetic Rat Heart

**DOI:** 10.7759/cureus.7095

**Published:** 2020-02-24

**Authors:** Ishfaq A Bukhari, Osama Y Mohamed, Abdulrahman A Almotrefi, Bassem Y Sheikh, Omnia Nayel, Fahim Vohra, Sibtain Afzal

**Affiliations:** 1 Pharmacology, College of Medicine, King Saud University, Riyadh, SAU; 2 Neurosurgery, College of Medicine, Taibah University, Almadinah Almunawara, Madinah, SAU; 3 Pharmacology, College of Medicine, University of Alexandria, Alexandria, EGY; 4 Prosthetic Dental Sciences, College of Dentistry, King Saud University, Riyadh, SAU; 5 Allergy and Immunology, College of Medicine, King Saud University, Riyadh, SAU

**Keywords:** olive oil, diabetes, ischemia/reperfusion induced cardiac arrhythmias, rat hearts

## Abstract

Background

Olive oil is rich in monounsaturated fatty acids and has been reported for a variety of beneficial cardiovascular effects, including blood pressure lowering, anti-platelet, anti-diabetic, and anti-inflammatory effects. Diabetes is a major risk factor for cardiac dysfunctions, and olive oil prevents diabetes-induced adverse myocardial remodeling.

Objective

The study aimed to evaluate the effects of olive oil against streptozotocin-induced cardiac dysfunction in animal models of diabetes and ischemia and reperfusion (I/R)-induced cardiac arrhythmias.

Methods

Diabetes was induced in male rats with a single intraperitoneal injection of streptozotocin (60 mg/kg i.p), rats were treated for five, 15, or 56 days with olive oil (1 ml/kg p.o). Control animals received saline. Blood glucose and body weight were monitored every two weeks. At the end of the treatment, rats were sacrificed and hearts were isolated for mounting on Langedorff’s apparatus. The effect of olive oil on oxidative stress and histopathological changes in the cardiac tissues were studied.

Results

The initial blood glucose and body weight were not significantly different in the control and olive-treated animals. Streptozotocin (60 mg/kg i.p) caused a significant increase in the blood glucose of animals as compared to saline-treated animals. The control, saline-treated diabetic animals exhibited a 100% incidence of I/R-induced ventricular fibrillation, which was reduced to 0% with olive oil treatment. The protective effects of olive oil were evident after 15 and 56 days of treatment. Diltiazem, a calcium channel blocker (1 µm/L) showed similar results and protected the I/R-induced cardiac disorders. The cardiac tissues isolated from diabetic rats exhibited marked pathological changes in the cardiomyocytes, including decreased glutathione (GSH) and increased oxidative stress (malondialdehyde; MDA). Pretreatment of animals with olive oil (1 ml/kg p.o) increased GSH and decreased MDA levels. Olive oil also improved the diabetic-induced histopathological changes in the cardiomyocytes.

Conclusion

Olive oil possesses cardiac protective properties against I/R-induced cardiac arrhythmias in rats. It attenuated oxidative stress and diabetes-induced histopathological changes in cardiac tissues. The observed cardiac protectiveness of olive oil in the present investigation may be related to its antioxidant potential.

## Introduction

Olive oil is the primary source of fat in the Mediterranean diet, which is associated with low morbidity and mortality for cardiovascular disease [1]. Olive oil, besides having a high level of monounsaturated fatty acids, contains other components with important biological properties [2]. Diabetes is a major risk for the development of cardiac dysfunctions such as cardiac fibrosis and atrial fibrillation [3-4]. A growing amount of evidence from preclinical and clinical studies has shown that olive oil improves insulin resistance, decreases vessel stiffness, and prevents thromboembolism [5-7]. The consumption of the Mediterranean diet, supplemented with olive oil, has been reported with a reduced rate of diabetes and its complications [8]. Extra virgin oil and its fractions possess antioxidant properties and have been shown to exhibit a cardiac protective effect in rat hearts [9]. Several studies have shown that the chronic administration of edible oils, including olive oil, reduced blood pressure, attenuated the loss of hypertension-induced left ventricular cardiomyocytes, and deceased adverse myocardial remodeling in spontaneously hypertensive and streptozotocin (STZ) diabetic rats [10-11]. The preventive role of olive oil against atrial fibrillations has been studied clinically [12]. Other edible oils, such as marine fish oil, prevented ischemia-induced ventricular fibrillation in rats [13]. The significance of olive oil is also well-established in Islamic medicine. Olive oil has a long, traditional use for many health benefits but scientific studies are still scarce to validate its traditional uses. STZ can cause pancreatic β-cell destruction; it is widely used experimentally as an agent capable of inducing insulin-dependent diabetes mellitus type 1 (T1DM). This model closely resembles human T1DM with chronic pancreatic islet inflammation, insulitis, and insulin deficiency [14]. The STZ-induced diabetic model is widely used to assess the therapeutic potential of new drugs in diabetes-induced complications such as heart problems, hypertension, thromboembolism, endothelial dysfunction, and dyslipidemia [15]. It has been reported that olive consumption decreases the risk of type 2 diabetes and associated complications [16]. Clinical study has revealed that the Mediterranean diet, which comprises olive oil, improves the postprandial glucose and has an antiatherosclerotic effect. Owing to the beneficial cardiovascular effects of edible oils against ischemia-reperfusion-induced cardiac arrhythmias and the diverse medicinal benefits of olive oil in cardiovascular disorders, the aim of our current project was to assess the effects of acute and chronic administration of olive oil against ischemia reperfusion-induced cardiac arrhythmias in isolated diabetic and non-diabetic rat hearts.

## Materials and methods

Animals

Male Wistar rats (200-250 g) were obtained from the Animal Care Center, College of Medicine, King Saud University, Riyadh, Saudi Arabia. The experimental protocol was reviewed and approved by the board of Council of Medical Research, College of Medicine, King Saud University, Riyadh, and complied with the National Institutes of Health guidelines for the care and use of laboratory animals. The animals were housed under standard laboratory conditions with a 12 h light: dark cycle with access to food and water ad libitum.

Chemicals

Extra virgin olive oil (Hintz foods production, Bremen, Germany) was purchased from the local market. All other chemicals and reagents used in the experiments were of analytical grade.

Induction of diabetes

Diabetes was induced by the method described elsewhere briefly; male rats (200-250 gm) were treated with freshly prepared streptozotocin (60 mg/kg i.p) [17]. Three days after the administration of streptozotocin injection, fasting glucose levels were measured. Animals with a fasting blood glucose (FBG) level of >300 mg/dl were included in the study. Rats were divided into different groups: control (saline-treated non-diabetes mellitus, NDM), control (NDM, olive-treated), control (saline-treated diabetes mellitus, DM), and DM treated with olive oil (1 ml/kg orally) for a period of five, 15, or 56 days. FBG, body weight, and blood pressure were monitored on a weekly basis. After 56 days of treatments with olive oil, the animals were killed and hearts were isolated and assessed for cardiac arrhythmias using the Langendorff apparatus.

Blood pressure measurement in conscious rats

Arterial blood pressures in the control and olive oil-treated rats were monitored weekly using the tail-cuff method using the MK2000 ST digital blood pressure monitor (Muromachi Kikai Co., Ltd., Japan). Baseline blood pressure was registered before the initiation of the treatment.

Ischemia-reperfusion-induced arrhythmia

The method described elsewhere was employed [18]. Hearts from male Wistar rats (250-300g, King Saud University breed) were perfused with Krebs-Henseleit solution (composition in mM: NaCl 11 8.4, KCl 1.3, MgSO_4_ 1.2, KH2PO_4_ 1.2, NaHCO_3_ 25.0, CaCl_2_^ ^2.5, glucose 11.5) gassed with 95% O_2_, 5% CO_2_ at a constant flow of 10 ml/ min and maintained at 37^0^C. Hearts were isolated from the olive oil-treated rats, a ligature was loosely sited around the left coronary artery at the point where the vessel emerged from under the left atrium.

Isometric contractions, at a resting tension of 2 g, were recorded via a small hook placed in the apex of the left ventricle and attached to a Harvard UFI transducer. Coronary perfusion pressure was monitored with a Washington PT400 pressure transducer. Surface electrical records were obtained from thin wire electrodes placed on the right atrium and apex of the left ventricle. All signals were fed into Harvard transducer interfaces and then into PowerLab/8sp (ADInstruments, Dunedin, New Zealand) connected to a computer. All hearts were allowed a 15 min stabilization period. Following the stabilization period, the coronary artery ligature was tightened and released after 10 min. Ventricular arrhythmic activity is recorded for three min post-ligation. Five parameters of ventricular arrhythmias were quantified during the ligation (10 min) and after the release of the ligature for 3 min: number of premature ventricular contractions (PVCs); percentage incidence of ventricular tachycardia (VT); duration of VT; percentage incidence of ventricular fibrillation (VF); and duration of VF.

Histological and biochemical assays

At the end of treatment with olive oil or vehicle, the ventricles of the heart were dissected, divided into two portions; one for detecting the underlying structural changes and the other for determining some of the concurrent functional alterations.

Structural Histological Examination

Heart specimens were fixed by 10% formol saline, processed, embedded, sectioned, and stained by hematoxylin-eosin, Masson's trichrome, or van Gieson stains for the detection of varied structural histopathological changes by light microscope.

Malondialdehyde (MDA) Assay

The principle is based on the reaction of MDA with thiobarbituric acid (TBA) (E. Merck Ltd., Bombay, India), according to the method described elsewhere [19]. The concentration of the MDA-TBA complex was being quantified, measured spectrophotometrically at 532 nm, and expressed as nmol/g wet tissue.

Reduced Glutathione (GSH) Assay

The principle is based on the reaction of reduced GSH with 5.5-dithiobis-(2-nitrobenzoic acid (DTNB) (E. Merck Ltd., Bombay, India), according to the method described elsewhere [20]. The absorbance was measured by the Shimadzu double beam spectrophotometer (UV200S; Shimadzu Corporation, Kyoto, Japan) at 412 nm. The amount of GSH present was calculated using a standard solution of GSH containing 1 mg of GSH/ml of 3% metaphosphoric acid. The increase in the extinction at 412 nm was proportional to the amount of GSH present and was expressed as nmol/g wet tissue.

Statistical analysis

All values are represented as mean ± SEM or percentage incidence. X2 analysis was used to test the data on the incidence of VT, VF. One-way analysis of variance (ANOVA) followed by the Newman-Keuls test was used for other data. *p< 0.05, **p< 0.01, and ***P< 0.001 indicated statistical significance. Statistical analysis was performed using Graphpad Prism version 5 (Sydney, Australia).

## Results

Induction of diabetes

**Figure 1:**
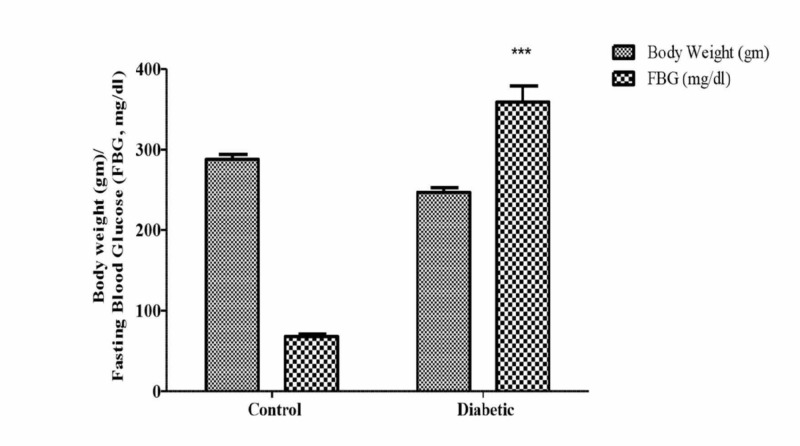
Comparison of body weight (g) and fasting blood glucose (FBG; mg/dl) in normal (control) and diabetic rats Values represent mean ± SEM (n=10), ***P<0.001 compared to control. Animals were treated with a single injection of saline or streptozotocin (60 mg/kg, i.p.).

As shown in Figure 1, the control rats treated with normal saline had a fasting blood glucose level of 68 ± 3 mg/dL. Animals that were administered a single i.p injection of streptozotocin (60 mg/kg) had a fasting blood glucose (FBG) level of 382 ± 22 mg/dL (P<0.001). The mean body weight of the control rats was 288 ± 6 gm while the mean body weight of diabetic rats was 247 ± 6 gm. 

Effect on mean blood pressure

**Figure 2:**
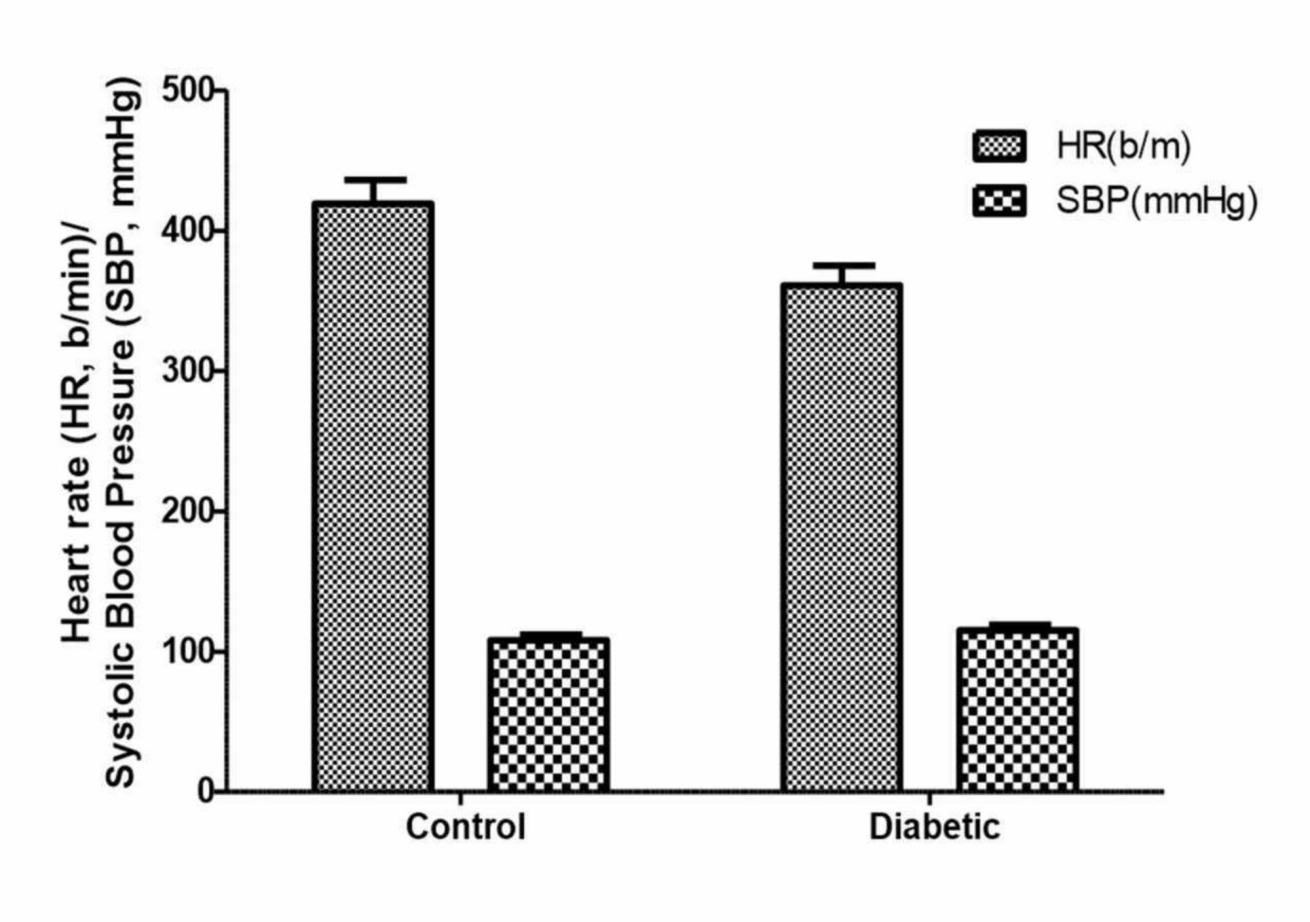
Effects of olive oil (1 ml/kg p.o) on heart rate (HR b/m) and systolic blood pressure (SBP; mmHg) in normal (control) and diabetic rats Values represent mean ± SEM (n=10) Animals received daily saline or olive ((1 ml p.o) for 15 days.

The mean blood pressure (MBP) of control rats was 108 ± 4 mmHg and diabetic rats had an MBP of 115 ± 5. Olive oil (1 ml p.o.) for 15 days treatment had no significant effect on blood pressure. The heart rate of diabetic rats was decreased as compared to control animals (Figure 2).

Effect on ischemia-reperfusion induced cardiac arrhythmia (I/R)

**Figure 3:**
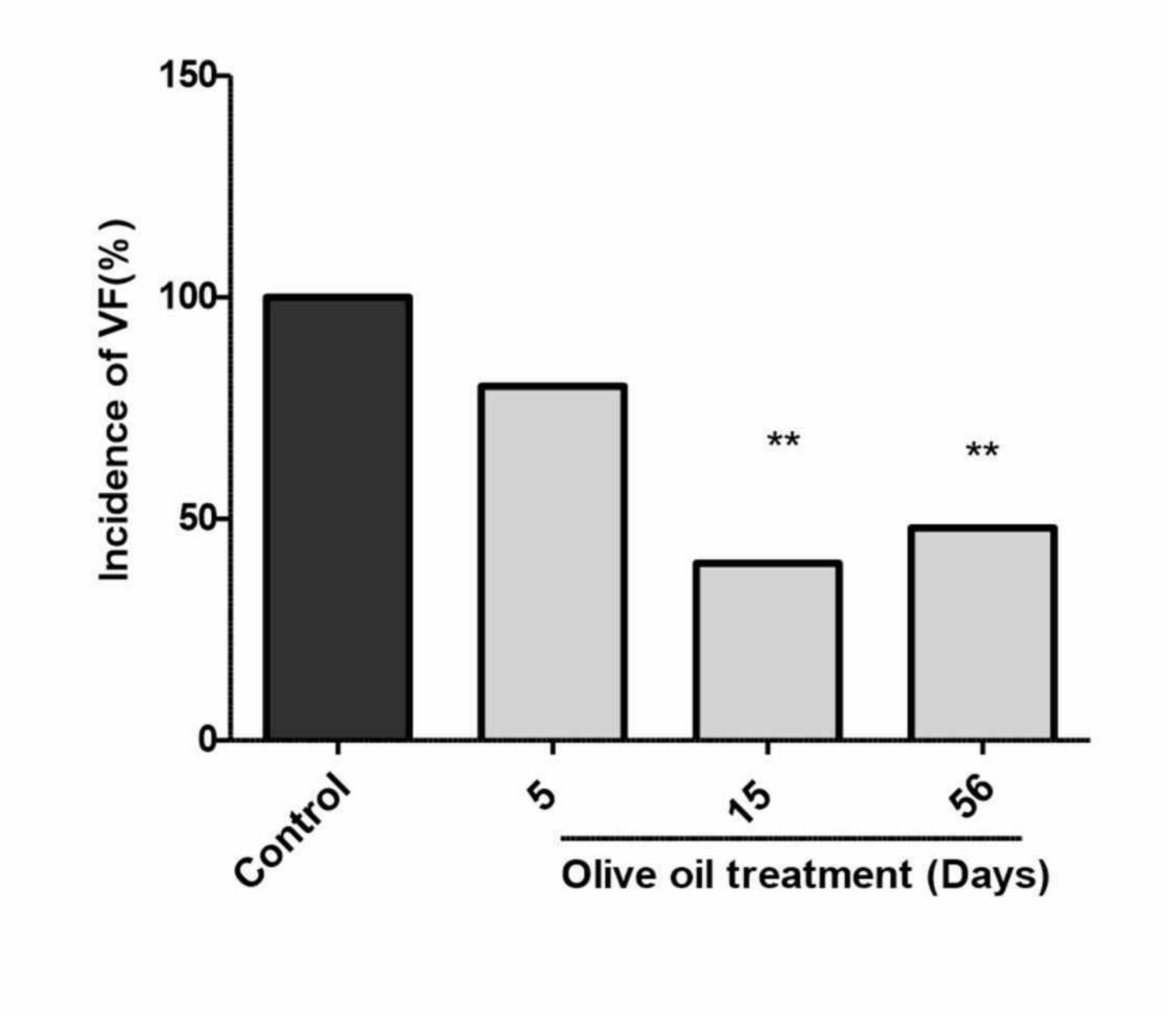
Effects of olive oil (1 ml/kg p.o) on the incidence of ischemia/reperfusion-induced ventricular fibrillation (VF) in isolated rat hearts (non-diabetic) Values represent the percentage of animals showing ventricular fibrillations (n=10). ** P<0.01 compared to control. Olive oil (1 ml/kg p.o.) was administered for five, 15, and 56 days.

**Figure 4:**
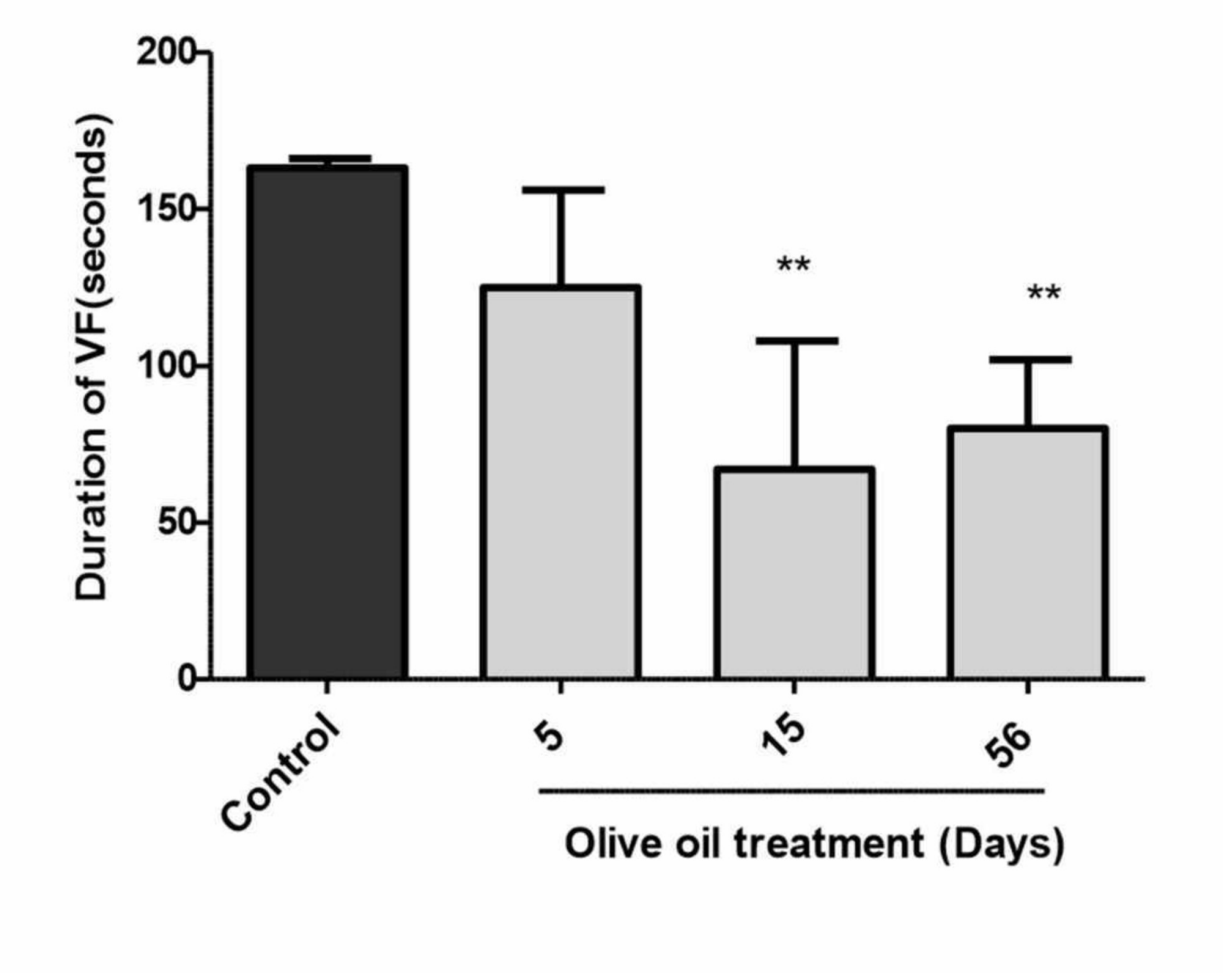
Effects of olive oil (1 ml/kg p.o) on the duration of ischemia/reperfusion-induced ventricular fibrillation in isolated rat hearts (non-diabetic) Values represent mean ± SEM of (n=10) duration of VF (sec). ** P<0.01 compared to control. Olive oil (1 ml/kg orally) was administered orally for five, 15, or 56 days.

**Figure 5:**
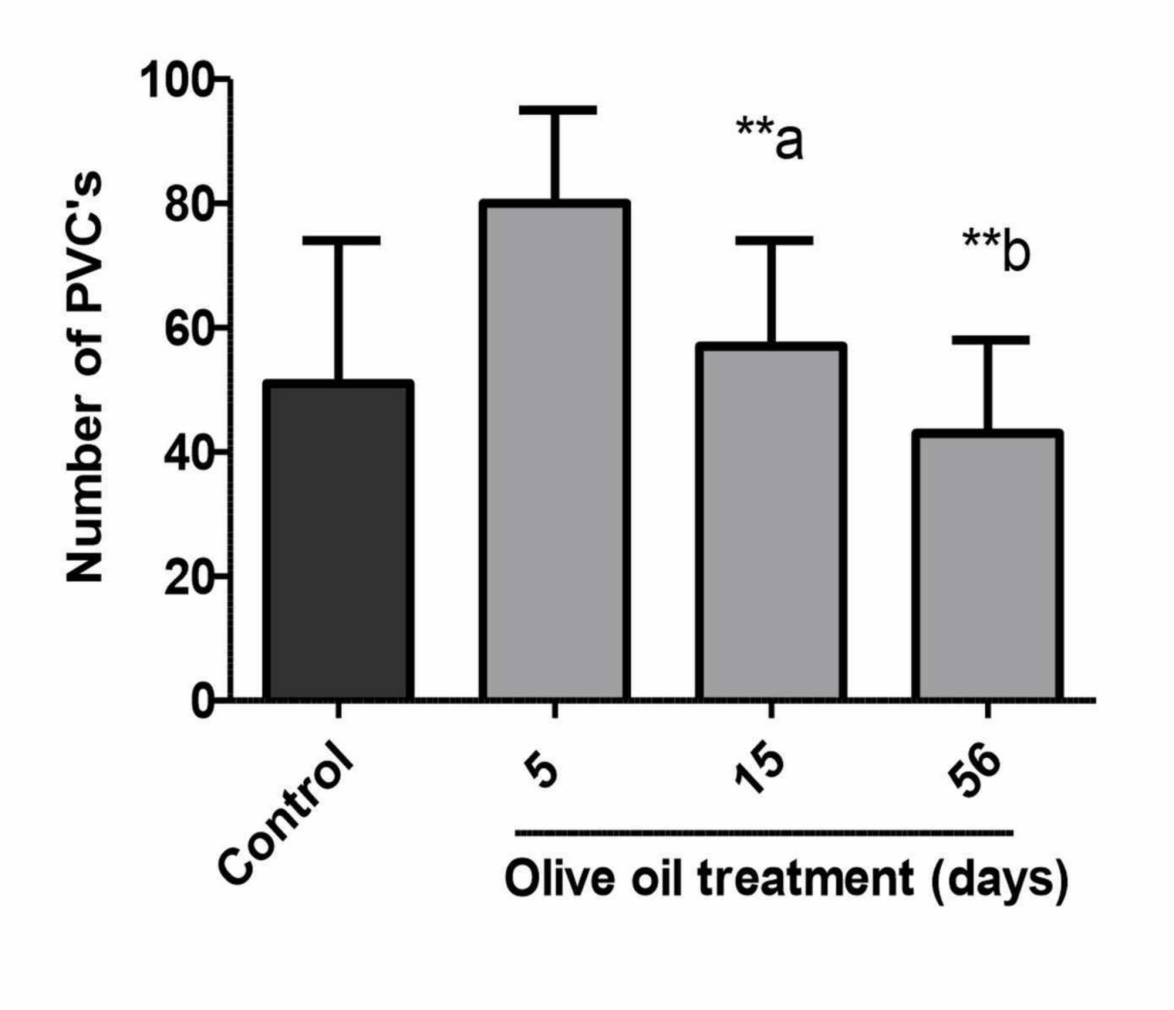
Effects of olive oil (1 ml p.o) on ischemia/reperfusion-induced number of premature ventricular contractions (PVCs) in isolated rat hearts (non-diabetic) Values represent mean ± SEM of (n=10) number of PVCs (n=10). **a. P<0.01 compared to the animal group treated for five days and **b. P<0.01 compared to control. Olive oil ( 1 ml/kg p.o.) was administered orally for five, 15, or 56 days.

As shown in Figures 3-4, in the non-diabetic control rats, the incidence and duration of ventricular fibrillation (VF) were 100% and 163 ± 3 seconds respectively. Pre-treatment of animals with olive oil (1 ml/kg, p.o.) for five to 56 days significantly decreased the incidence and duration of VF. However, a statistically significant (P<0.01) decrease in incidence and duration was obtained after 15 and 56 days of treatment with olive oil (Figures 3-4). There was no appreciable difference in the heart rate and perfusion pressure of control and olive oil-treated animals; however, the heart rate of animals treated with olive oil was decreased as compared to saline-treated animals (data not shown). There was a variable effect of olive oil treatment on the premature ventricular counts (PVCs) (Figure 5).

**Figure 6:**
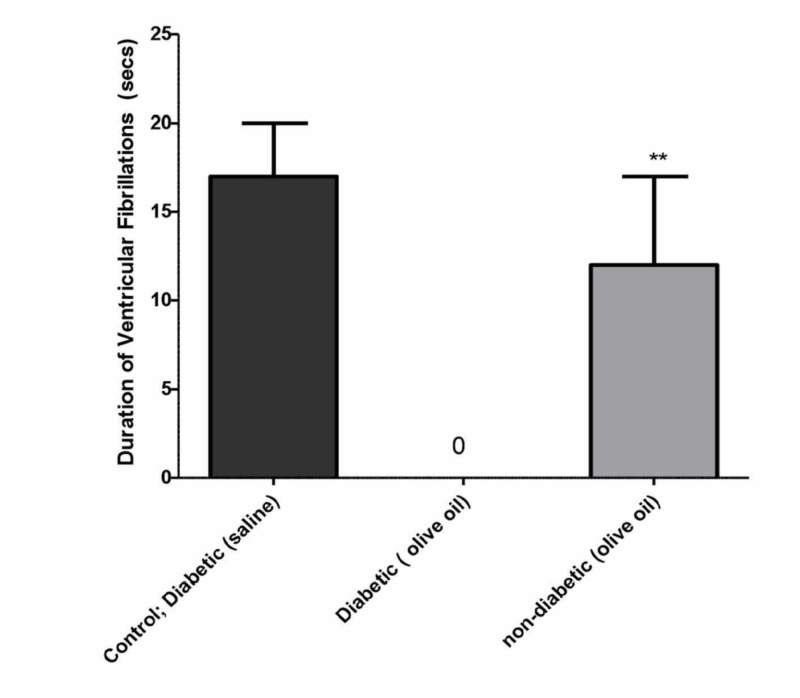
Effects of olive oil (1 ml/kg, p.o) on the duration of ischemia/reperfusion-induced ventricular fibrillation in isolated rat hearts in control (diabetic; saline treated), diabetic (olive oil treated), and non-diabetic (olive oil treated) animals Olive oil (1 ml/kg p.o) was administered orally for 56 days. None of the diabetic rats treated with olive showed ventricular fibrillation (VF). Values represent the percentage of animals showing ventricular fibrillation (n=10). ** P<0.01 compared to control

**Figure 7:**
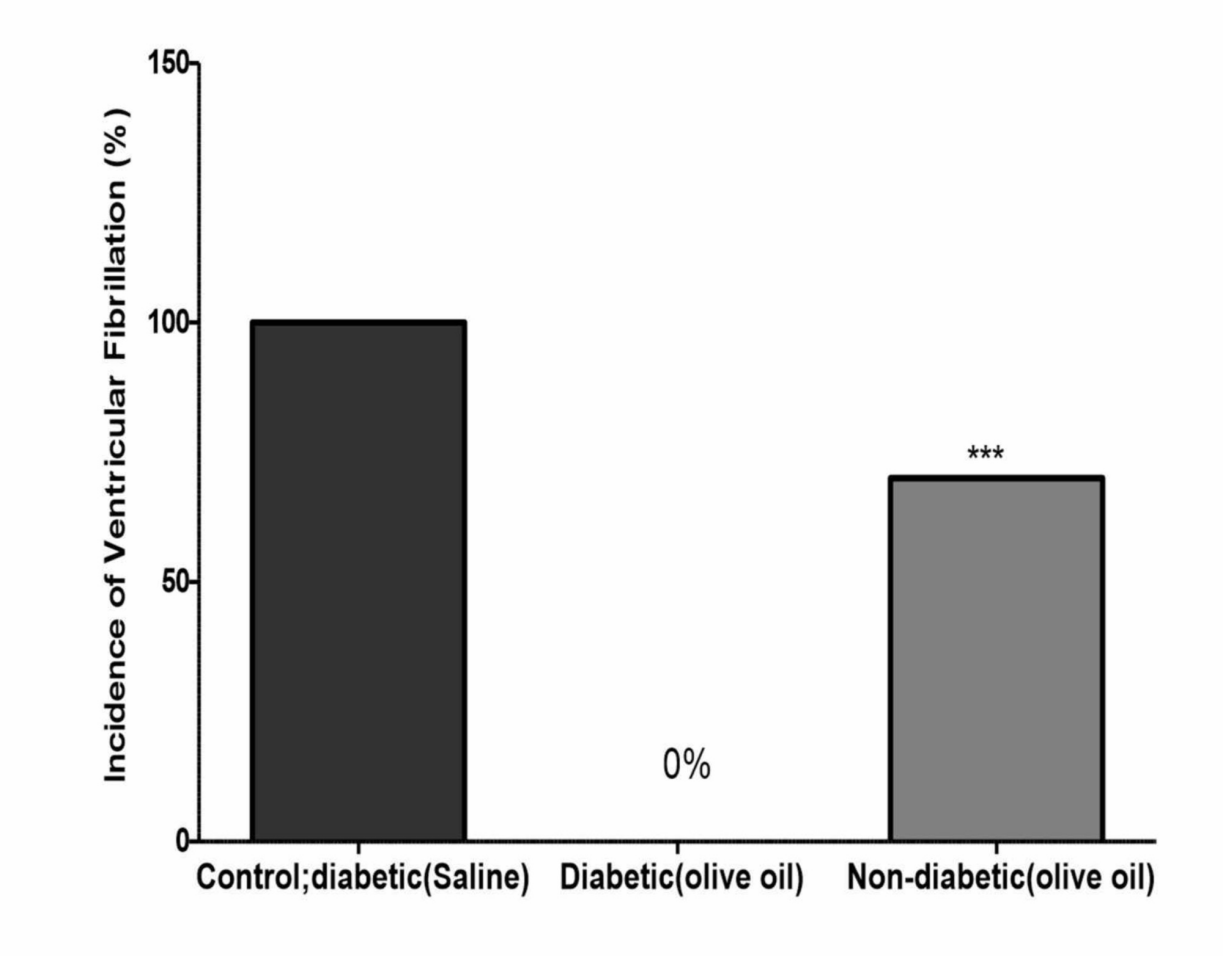
Effects of olive oil (1 ml p.o) on the incidence of ischemia/reperfusion-induced ventricular fibrillation in isolated rat hearts in control diabetic (saline), diabetic (olive oil treated), and non-diabetic (olive oil treated) Values represent the percentage of animals showing ventricular fibrillation (VF) (n=10). *** P<0.001 compared to control. Olive oil was administered orally for 56 days. None of the diabetic rats treated with olive oil showed VF.

Similar to non-diabetic control animals, the incidence of VF was 100% in diabetic rats, which was reduced to 0% with olive oil treatment for 56 days. The incidence of VF was reduced to 70% in the non-diabetic rats treated with olive oil for 56 days (Figure 6). Similarly, the duration of VF was completely suppressed (Figure 7). Diltiazem (1 µM/mL), used as the reference drug, produced complete inhibition of both ischemia and reperfusion-induced cardiac arrhythmias (data not shown).

Reduced glutathione (GSH) assays

**Figure 8:**
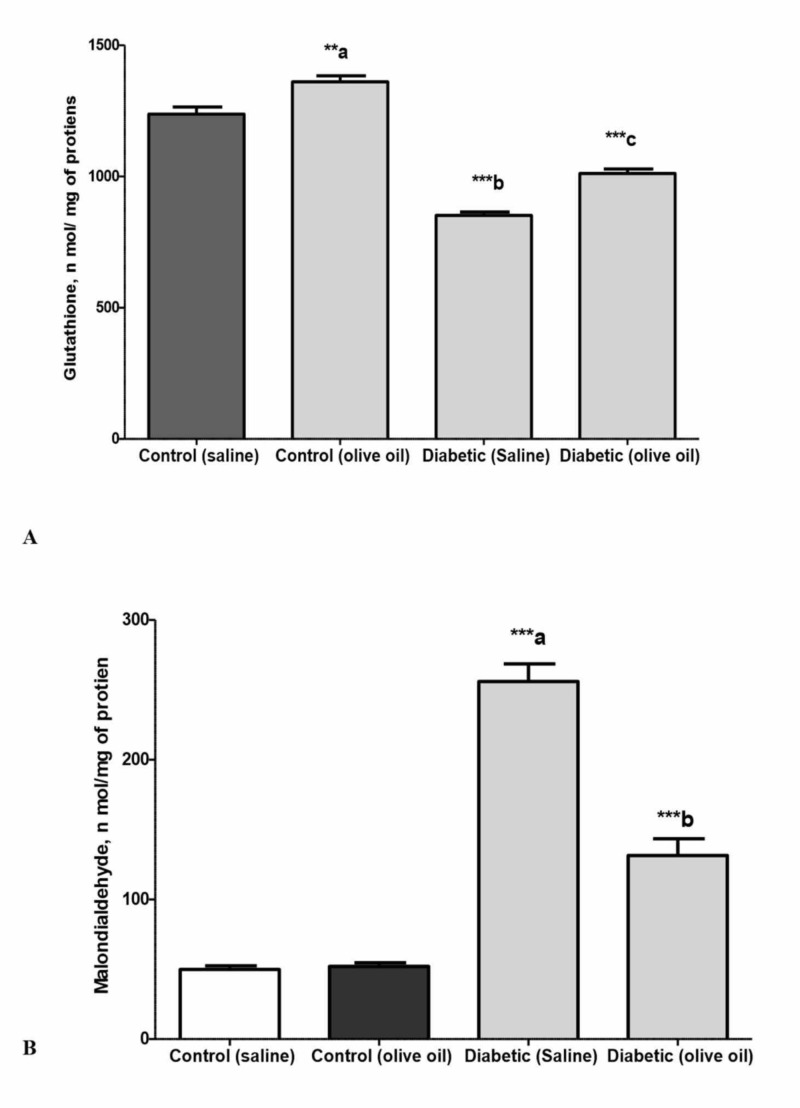
Levels of glutathione (A) and malondialdehyde (B) in isolated cardiac tissues of the control and diabetic rats treated with saline or olive oil (1 ml/kg p.o.) for 56 days Data represent mean ± SEM (N=8). **a p<0.01, ***a P<0.001 compared to control saline treated, ***b P<0.001 compared to diabetic saline treated, and ***c P<0.001 compared to diabetic saline treated

As shown in Figure 8A, the GSH contents of isolated heart specimens obtained from the non-diabetic control (saline) and control (olive oil) groups were significantly higher (P<0.001) compared to the diabetic saline-treated animals (Figure 8A). As shown in the figure, the pretreatment of animals with olive oil (1 ml/kg p.o) caused a significant increase in the GSH content of heart specimens obtained from the diabetic olive-treated group (P<0.001).

Malondialdehyde (MDA) assay

The MDA contents of isolated heart specimens obtained from the control (saline-treated) or control (olive oil-treated) rats were significantly lower (P<0.001) as compared to the diabetic saline-treated group (Figure 8B). The content of MDA in the diabetic olive oil-treated group was significantly reduced (P<0.001) as compared to the diabetic saline-treated group. There was no significant difference in the MDA contents of the control saline-treated and control olive oil-treated groups (P<0.001) (Figure 8B).

Histological analysis

**Figure 9:**
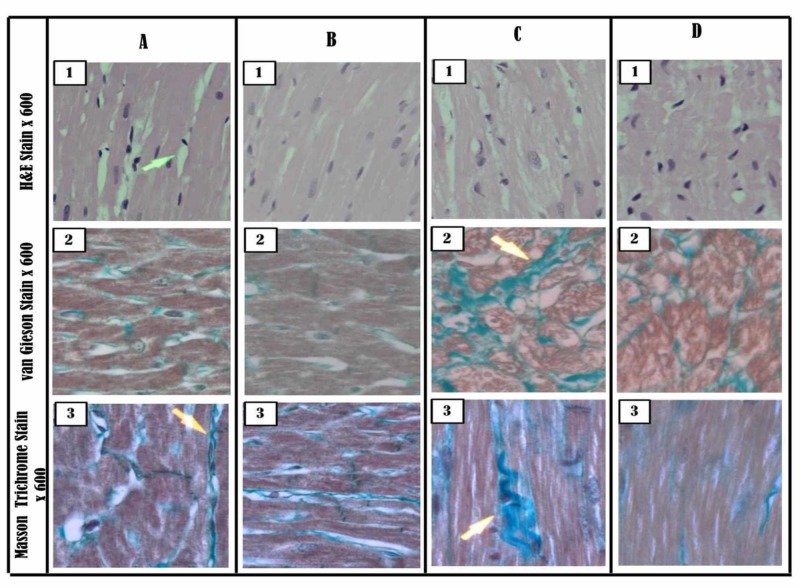
Representative images of the histopathological analysis of isolated heart tissues of normal control rats and diabetic rats treated with saline and olive oil (A) Control saline-treated; A 1: normal myocardial fibers with mild interstitial edema; A 2 & 3: normal myocardial connective tissue distribution (B) Control olive oil-treated; B 1: normal myocardial fibers; B 2 &3: normal collagen distribution (C) Diabetic control; C 1: enlarged pleomorphic nuclei with swelling and disruption of myocardial fibers; C 2 & 3: extensive interstitial fibrosis with bands of dense collagen strands (D) Diabetic olive oil-treated; D 1: absence of enlarged pleomorphic nuclei, though minimal cellular edema and myocardial fiber disruption is still apparent; D, 2 & 3: reduction in the amount of interstitial fibrosis Animals were treated with saline or olive oil (1 ml/kg p.o) for 56 days.

Figure 9A shows the histopathological changes in the specimens of hearts isolated from normal rats. There was mild interstitial edema in myocardial fibers, with normal myocardial connective tissue distribution (Figure 9A 2&3). Non-diabetic rats fed on olive oil for 56 days had no changes in the myocardial fibers (Figure 9B 1) and normal collagen distribution (Figure 9B 2&3):

As shown in Figure 9C 1, heart tissues from diabetic animals had enlarged pleomorphic nuclei with the swelling and disruption of myocardial fibers. Figure 9C 2&3 shows there was extensive interstitial fibrosis with bands of dense collagen strands. On the other hand, diabetic animals treated with olive oil exhibited the absence of enlarged pleomorphic nuclei, though there was minimal cellular edema of cardiomyocytes (Figure 9D 1) with a reduction in the amount of interstitial fibrosis (Figure 9D 2&3).

## Discussion

The Mediterranean diet is associated with low mortality for cardiovascular disease due to its olive oil content [5,21]. Olive oil is a rich source of monounsaturated fatty acids and other components with proven beneficial effects on the cardiovascular system [21-22]. In the present study, we assessed the beneficial cardiac effects of olive oil in streptozotocin-induced diabetic rat models. Baseline experiments were conducted in normal rats to establish the dose and duration of olive oil therapy. Animals treated with saline exhibited an increased incidence of cardiac arrhythmias when tested in I/R-induced cardiac arrhythmias in isolated rat hearts. The pretreatment of animals with olive oil for five to 56 days caused a marked reduction in the incidence and duration of VF. The maximum effect was observed on the 56th day of the treatment; therefore, this duration of treatment was chosen for further studies on diabetic rats. Diabetes was induced by a single intraperitoneal injection of streptozotocin. STZ causes pancreatic β-cell destruction and is a widely used experimental tool to assess the therapeutic potential of new drugs in diabetes-induced complications such as heart problems, hypertension, thromboembolism, endothelial dysfunction, and dyslipidemia [15]. STZ is capable of inducing insulin-dependent diabetes type 1 diabetes mellitus (T1DM), which closely resembles the human T1DM, with chronic pancreatic islet inflammation and insulin deficiency [14]. Diabetic control rats treated with saline had a marked increase in the incidence of ventricular fibrillation. Previous studies have shown that diabetes is a major risk factor of oxidative stress that leads to cardiovascular disorders, including cardiac arrhythmias [4].

Oxidative stress contributes mainly to the pathogenesis of diabetes complications [23]. Normally, free radical superoxide anions generated in the body are rapidly scavenged by natural cellular defense mechanisms, such as superoxide dismutase (SOD) and catalase (CAT), and by the antioxidant action of GSH [24-25]. There is a tremendous increase in oxidative stress and a decrease in the free radical scavenging capability of the body in diabetes mellitus that leads to diabetic complications [26]. The protective effect of olive oil against diabetes-induced cardiac arrhythmia may have occurred by decreasing oxidative stress in the cardiac tissues. This hypothesis was further strengthened when cardiac tissues from olive oil-treated animals exhibited a significant increase in GSH levels and a decrease in MDA levels. MDA is one of the secondary products of lipid peroxidation and is a well-established potential biomarker for oxidative stress [27]. MDA concentration has been an indicator of oxidative imbalance during the onset of several diseases [28]. In the present study, the GSH levels were decreased and the MDA levels were increased in the cardiac tissues isolated from diabetic rats, which was consistent with earlier reports. This, in turn, will lead to increased oxidative stress and cardiac injury in diabetic rats [25]. The decreased level of GSH and increased MDA levels have been reported to be positively correlated with I/R-induced damage [29]. Olive oil is rich in antioxidant components (enzymatic and non-enzymatic) such as catalase, superoxide dismutase, reduced glutathione, ascorbic acid, and reduced nicotinamide adenine dinucleotide phosphate (NADPH)-recycling dehydrogenase [30]. The cardiac protective effect of olive oil may be attributed to these components. Histopathological analysis of cardiac tissues showed that diabetic untreated animals had enlarged pleomorphic nuclei with swelling and disruption of myocardial fibers and there was extensive interstitial fibrosis with bands of dense collagen strands. These histopathological changes were reversed in diabetic animals treated with olive oil, indicating that olive oil prevents diabetes-induced pathological changes in the heart. These findings validate the traditional uses of olive oil in prophetic medicine and the lower incidence of cardiovascular events in people using the Mediterranean diet.

## Conclusions

The present study investigated the effects of olive oil against streptozotocin-induced cardiac disorders using animal models of diabetes and ischemia and reperfusion (I/R)-induced cardiac arrhythmias. Streptozotocin-induced diabetes increased the risk of incidence of I/R-induced ventricular fibrillation possibly by increasing oxidative stress in cardiomyocytes. The pretreatment of animals with olive oil prevented these deleterious cardiac changes. The cardiac protective effect of olive oil is most likely mediated via decreasing oxidative stress in the cardiac tissues. These findings validate the traditional use of olive oil for beneficial cardiovascular effects.
